# *Drosophila melanogaster* Fly Odors Alone are not Sufficient to Attract other Flies

**DOI:** 10.1007/s10886-026-01747-x

**Published:** 2026-08-06

**Authors:** Anjana Pisharody Unni, Karen Rihani, Silke Sachse, Bill S. Hansson, Markus Knaden

**Affiliations:** 1 Department of Evolutionary Neuroethology, Max-Planck Institute for Chemical Ecology, Jena, Germany; 2 Chair for Neurobiology and Genetics, Biocenter, University of Würzburg, Würzburg, Germany

## Abstract

Pheromone-mediated behaviors are crucial for the survival and reproduction of *Drosophila melanogaster*, governing activities such as courtship, mating, and aggression. While many studies have focused on short-range interactions involving direct contact between flies, the role of olfactory cues alone in this attraction remains poorly understood. In this study, we investigated the behavioral responses of virgin male and female *D. melanogaster* to conspecific headspace using a Y-maze. We tested fly responses under various environmental conditions (e.g., in the presence or absence of food odors and with or without social isolation of the tested animals before the experiments). Finally, we repeated some of the experiments in a still-air trap assay. Our results suggest that fly odors may play only a limited role in mate-seeking behavior in *D. melanogaster*.

**Supplementary Information:**

The online version contains supplementary material available at https://doi.org/10.1007/s10886-026-01747-x.

## Introduction

To survive the evolutionary race, insects must interpret multiple sensory inputs and respond optimally to environmental cues. In *Drosophila melanogaster*, sensory inputs are processed through various modalities like e.g. vision, olfaction, gustation (Currier and Nagel 2018; Oh et al. 2021). In a social context, the flies use pheromones, i.e. chemicals, detected either through olfaction in case of air-borne chemicals or through gustation in case of non-volatile contact chemicals (Kohl et al. 2015). These pheromones are pivotal in many key behaviors such as courtship, mating, aggression, and aggregation (Fernández and Kravitz 2013; Wang and Anderson 2010).

*Drosophila* pheromones have been extensively studied, and continue to be actively investigated. Many pheromones are produced predominantly by one sex and act in combination with other chemical cues to elicit specific behavioral responses. For instance, female-specific compounds such as 7,11-heptacosadiene and 7,11-nonacosadiene along with other pheromones enhance female attractiveness to males (Antony and Jallon 1982; Marcillac et al. 2005). In some cases, a single pheromone can have multiple roles acting as an essential part of a pheromone blend eliciting different behaviors in different contexts. For example 11-cis-vaccenyl acetate (cVA), a well-studied pheromone produced in male ejaculatory bulbs (Butterworth 1969) and transferred to females during copulation, has multiple behavioral functions (Ejima 2015). In males, cVA acts as an anti-aphrodisiac (Zawistowski and Richmond 1986) and promotes male-male aggression (Wang and Anderson 2010), whereas in virgin females, it increases sexual receptivity (Kurtovic et al. 2007). When applied to food, cVA can increase the food’s attractiveness to females in a wind tunnel assay (Bartelt et al. 1985; Cazalé-Debat et al. 2019; Tolassy et al. 2023). In flies reared in isolation, acute exposure to cVA can trigger strong aggression (Liu et al. 2011). cVA released by ovipositing females governs joint oviposition (Duménil et al. 2016). These examples show that, pheromonal communication is highly dynamic and the response depends not only on the pheromone, but on the context, the pheromone combination, the background in which the animal detects the pheromone and other spatial parameters.

Despite extensive research on pheromone-mediated behaviors, most of these studies often focus on contact assays. Fly interactions are often studied in small circular arenas with a diameter of 1–2 cm and a height of less than 0.5 cm (Gil-Martí et al. 2023; von Philipsborn et al. 2023).

Studies on olfactory responses over distances in most cases rely on synthetic pheromones, often in the presence of background food odors, to study flight behavior (Becher et al. 2010; Borrero-Echeverry et al. 2022; Lebreton et al. 2015). However, interestingly, food previously been exposed to flies before became more attractive to conspecifics in both a wind tunnel assay (Cazalé-Debat et al. 2019) and in a field experiment (Wertheim et al. 2006), showing that flies can label food with compounds that attract other flies over a distance. Such assays again used food odors, making it difficult to narrow down the role of fly-emitted odors in these behaviors.

Here we ask whether flies themselves (i.e., not their olfactory remains at feeding places) attract other flies by olfactory cues. The challenge of identifying compounds of low volatility, such as cVA, in the headspace of virgin males (Everaerts et al. 2010) raises uncertainty about the role of naturally emitted volatiles in shaping distance-dependent behavioral responses in flies. At the same time, the recognition that odor perception differs between flying and walking flies (Stupski and Breugel 2024), together with an increasing effort to examine olfactory perception under conditions involving airflow and over longer distances (Suver et al. 2023; Tolassy et al. 2023) highlights the need to investigate fly behavior in different contexts. Hence, to contribute more to this particular field of research, we investigate whether headspace volatiles from groups of living male or female flies could attract individual flies over a short distance and with constant airflow.

The primary objective of this study was to determine whether virgin male or female flies are attracted to the headspace of living conspecifics. For this purpose, we placed virgin males or females as the odor source in one arm of a Y-maze with air flow and tested the response of individual flies to the headspace volatiles. We also measured the CO₂ concentration in the Y-maze to ensure that the levels were not too high due to high number of flies as the odor source. Additionally, we also conducted still trap assays to evaluate the response in still air. We hypothesized that olfactory cues emitted by a group would be sufficient to elicit a response in an individual fly. Specifically, we expected that virgin females would be attracted to male headspace, while virgin males would be attracted to female headspace. Additionally, given the known role of cVA in eliciting male aggression, we expected that virgin males would be repelled by male headspace.

To further explore the factors influencing olfactory-driven behavior, two additional experimental scenarios were tested. First, we examined the behavioral responses of flies that were kept in social isolation before the experiment. Social isolation, a passive stressor, has been shown to modulate behavioral responses to conspecifics (Vora et al. 2022), with stronger effects reported in males than in females (Leech et al. 2017; Liu et al. 2011). Second, we tested the response to the headspace of flies when blended with food odor. Since food odors can alter the odor profile of flies (Lin et al. 2015) and influence their response to conspecific odors (Bartelt et al. 1985; Cazalé-Debat et al. 2019; Das et al. 2017), we aimed to assess whether the presence of food modifies the behavior of tested flies.

## Materials and Methods

### Fly Stock

All fly stocks were maintained on a standard cornmeal-agar-molasses medium under a 12:12 light-dark cycle, with relative humidity (RH) at 70% and a temperature of 25 °C. For 1 L of the standard food, the following ingredients were used: beet syrup (118 g), brewer’s yeast (11 g), agar powder (4.1 g, Roth), hot water (540 mL), corn grits (95 g, Seitz), propionic acid (2.4 mL, Roth 99.5%), Nipagin 30% (3.3 mL, Sigma-Aldrich 99%), and cold water (378 mL). Only *Drosophila melanogaster* of the Canton-S strain were used. Flies were sexed on the day of adult eclosion and separated into vials containing 10 males or 10 females per vial. For experiments involving isolated flies, each fly was sexed and housed individually in a separate vial. Sexing of the flies was done using CO₂ anesthesia. All experiments were conducted using flies aged 4–7 days after adult eclosion. Flies used as the odor source for headspace experiments were also grouped in vials containing 10 flies of the same sex.

### Y-maze with Airflow

**Fig. 1:**
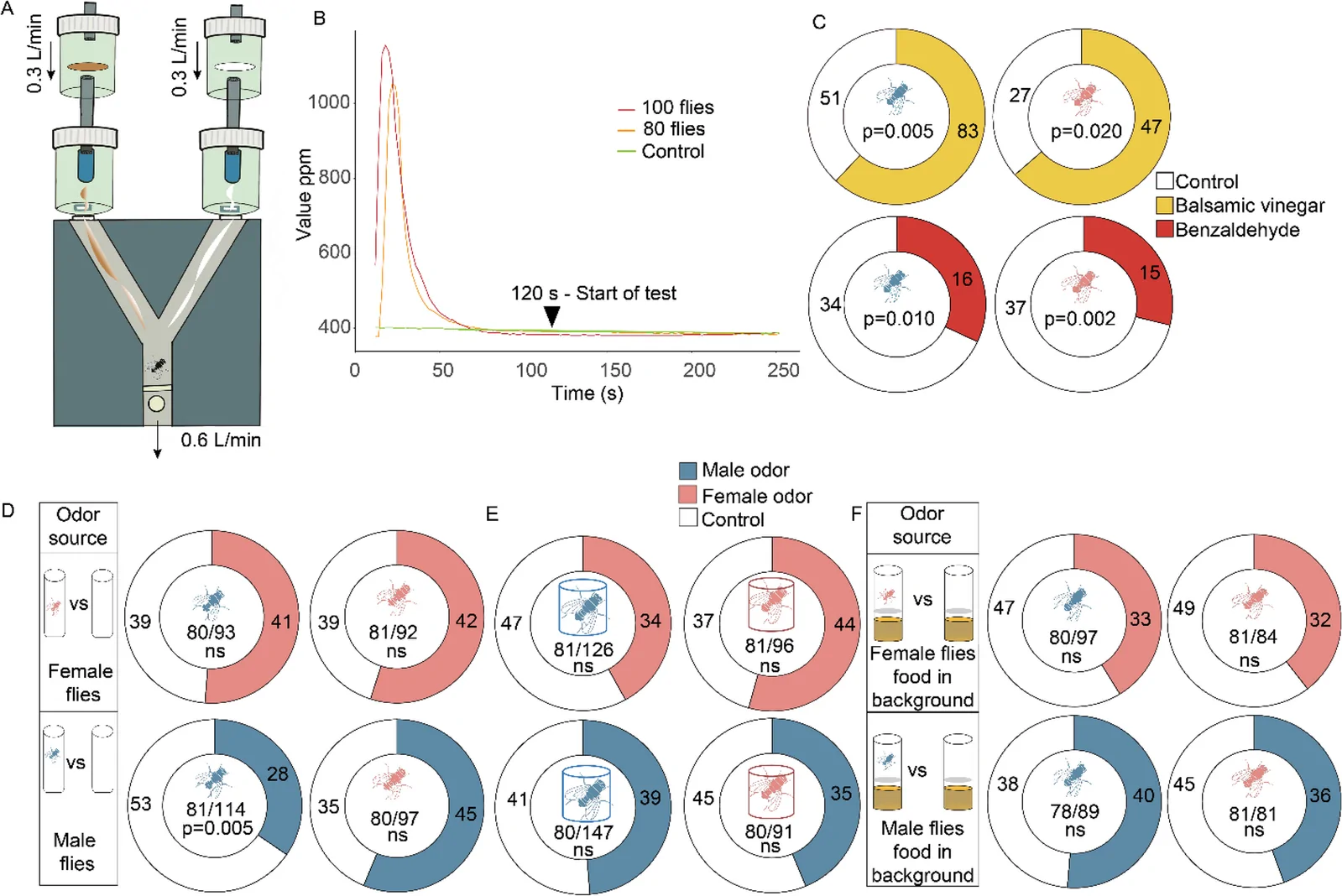
**A** Y-maze paradigm; arrows indicate direction of airflow through vials containing an odor source (either odor or living flies) through the Y maze. Equal airflow through both arms is established by sucking out 0.6 L/min from the center arm and allowing an influx of 0.3 L/min into each side arm. **B** Concentration of CO₂ measured in the Y maze entrance. **C-F** Results of Y-maze assay. Flies in the middle of the donut represent individually tested flies (blue, male flies; pink, female flies). Colored part of donut represents proportion of flies that preferred tested odor over clean-air control (white part of donut). **C** Response of males and females to the attractant balsamic vinegar (yellow donuts) and the repellent benzaldehyde (red donuts). **D** Response of males and females to the odors from virgin females (pink donuts) and virgin males (blue donuts). **E** Responses of flies reared in isolation. **F** Responses when food odor was added to fly headspaces and to empty controls. All significance levels are based on the chi-square goodness-of-fit test. Numbers within donuts depict the number of tested flies and the number of flies that made a choice in the Y-maze

The choice assay was conducted in a Y-maze connected to an airflow system supplying filtered and humidified air (Fig. 1A). The airflow was set to 0.3 L/min in tubes with an inner diameter of 4 mm. While the setup itself is small in size, the controlled airflow ensures that flies respond to volatiles carried over distance in a directional manner, rather than to odors and other sensory inputs encountered through proximity or contact. To validate the functionality of the Y-maze, the known attractant balsamic vinegar (200 µL, diluted 1:10 in distilled water) (Becher et al. 2010) and the known repellent benzaldehyde (200 µL, ACROS organics 99.5% purity diluted 1:10 in distilled water) (Knaden et al. 2012) were tested against distilled water. These tests resulted in clear attraction and aversion, respectively (Fig. 1C).

### Tests with Fly Headspace

For headspace tests, 80–100 virgin flies (either males or females) were placed in a vial serving as the odor source, which was then connected to one arm of the Y-maze. The second arm was connected to an identical but empty vial as a control. Airflow was initiated after setting up the vials.

In experiments testing fly headspace in the presence of food odors, the vial containing flies (odor source) and the control vial were both connected to separate food-containing vials, ensuring the flies had no direct access to the food. We used about 10 grams of unused fly standard food as the food source (for the recipe please see above). In one arm, airflow first passed over the food source, picking up food-related volatiles, and then passed over the fly headspace. In the control arm, airflow again first passed over the food source but was afterwards led through an empty vial. Hence, the flies could choose between the arm that had a combination of fly and food headspace, and just the food headspace. 

After two minutes, an individual fly was introduced into the entrance arm of the Y-maze. After an additional 30 s, a slit at the decision point was opened, allowing the fly to choose between the fly headspace and the control. The selected side and the time taken to make a decision were recorded. Flies that failed to make a choice within 5 min were excluded from the analysis. Each fly was tested only once. After completion of half the trials for each experiment, the Y-maze was washed and flipped, and the remaining trials were conducted thereafter.

### Still Air Trap Assays

**Fig. 2:**
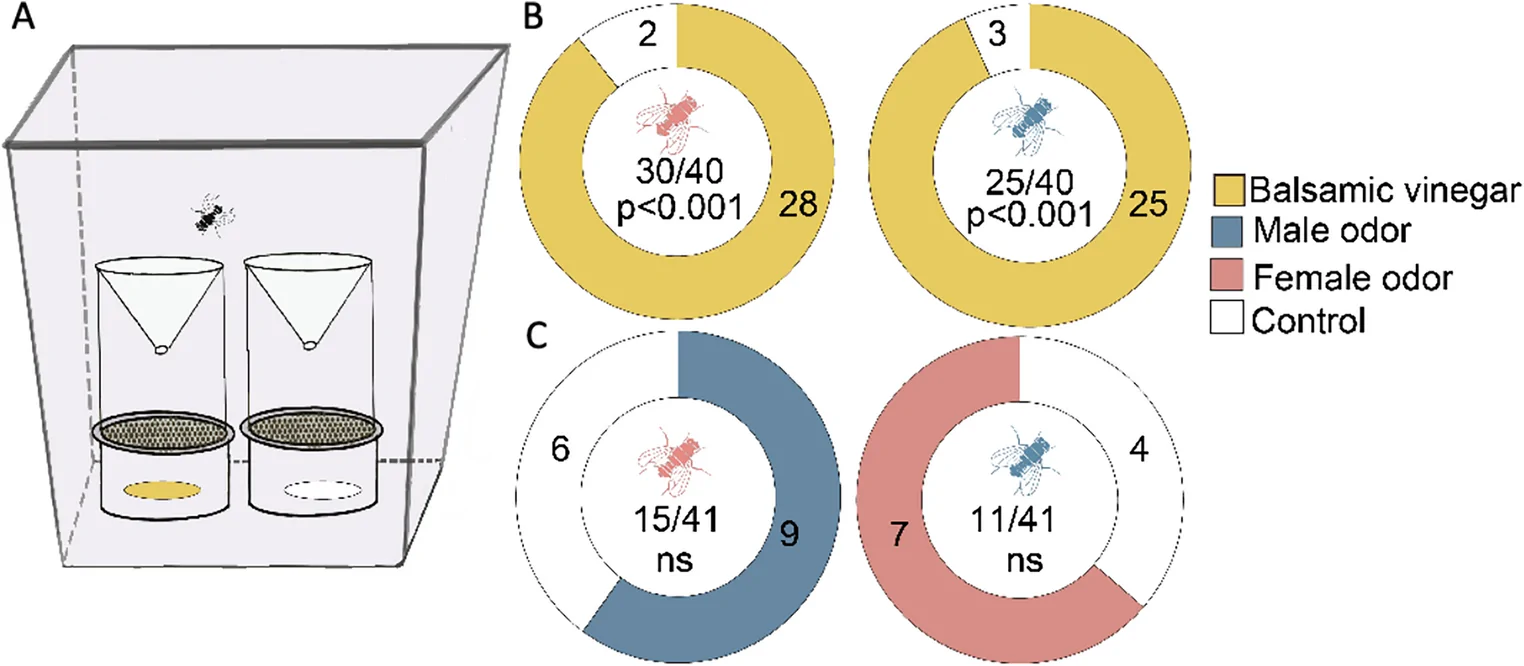
**A**. Trap assay. **B**. Responses of individual male (left, blue) and individual female (right, red) flies to the attractant balsamic vinegar (yellow donuts). **C**. Responses of individual females and males tested with headspace of 30 flies from the opposite sex. All significance levels are based on the chi-square goodness-of-fit test

To complement the Y-maze experiments, a still-air trap assay was conducted to evaluate responses to fly headspace in the absence of airflow. The trap assay was performed in transparent plastic salad boxes (~ 10 cm high, 5 cm long, 7 cm wide) with small air holes to allow air circulation while preventing fly escape. Each trap contained two vials: one housed living flies (either 30 virgin females or 30 virgin males) with a water source, while the other served as an empty control contained water alone. Positioned above each vial, separated by a metal mesh was an upper chamber where the test fly was captured upon entry. Flies entered the trap through a conical inlet made with paper that permitted entry but prevented exit, ensuring unidirectional trapping (Fig. 2A). Each trap was used only once. This assay has been previously validated for odor testing (Knaden et al. 2012), with balsamic vinegar serving as a positive control (Fig. 2B). The preference of the tested individual flies for the flies-containing vial versus the empty vial was recorded. The numbers of flies used as odor source differed between still-air and Y-maze assays to reflect differences in odor dilution and accumulation. Continuous airflow in the Y-maze requires larger fly groups (80–100) to maintain sufficient odor concentrations, whereas in the still-air trap assay, volatiles accumulate in the absence of airflow, suggesting reliable responses with fewer flies (30).

### Carbon Dioxide Emission Test

Flies are known to emit carbon dioxide (CO₂), which at high concentrations can act as a repellent (Suh et al. 2004). To determine whether the headspace contained behaviorally relevant concentrations of CO₂, we measured CO₂ emissions of 90–100 flies using a GMP251 CO₂ probe connected to a USB DAQ 6008 interface and analyzed the data using LabVIEW software (Fig. 1B).

### Statistical Analysis

For both the Y-maze and trap assays, only flies that made a choice were included in the statistical analysis. A chi-square goodness-of-fit test was performed to evaluate the distribution of choices. Paired t-tests were conducted to compare decision times between experimental conditions. All statistical analyses were performed using R (version 4.2.3, 2023-03-15 UCRT). The difference in time to make the choice was analyzed using a generalized linear model (GLM) for positive skew, including sex, social condition, and their interaction as fixed factors. Significance was assessed using likelihood ratio tests (analysis of deviance), and post hoc pairwise comparisons were performed using estimated marginal means with Tukey-adjusted p-values.

## Results

When testing the valence of virgin female headspace in the Y-maze, neither virgin males nor virgin females exhibited any significant response (Fig. 1D, pink donuts). In contrast, the headspace of virgin males, was neutral to virgin females, significantly repelled virgin males (Fig. 1D, blue donuts). Since no elevated carbon dioxide levels were detected in the airflow emanating from vials containing male flies (Fig. 1B), it is likely that this repellency was not caused by CO₂ but rather other male-derived odors in the headspace.

To further investigate whether social isolation affects fly responses to headspace odors, we tested flies that were reared in isolation. These isolated flies showed neither aversion nor attraction towards any of the headspaces tested (Fig. 1E). Interestingly, isolated flies took significantly longer than group-reared flies to make a decision (Figure S1). These findings suggest that social isolation impacts decision-making behavior in flies.

Next, we examined whether fly headspace could enhance the attractiveness of food headspace presented in the background. However, the presence or absence of fly headspace did not influence their original choices, suggesting that fly headspace does not significantly modulate attraction to food headspace under the tested conditions (Fig. 1F). We, however, acknowledge that carrying food odors over living flies may have altered the flies’ chemical emissions, and thus potentially represents a limitation of the present study.

Finally, we tested whether fly odors could elicit attraction or aversion in a still-air environment using a trap assay. To validate the setup, we first tested the response of individual male and female flies to the known attractant balsamic vinegar. Both sexes showed strong attraction to the balsamic vinegar trap (Fig. 2B), confirming the functionality of the assay. However, when we tested individual flies against traps containing 30 virgin flies of the opposite sex, no significant attraction was observed (Fig. 2C). The finding that neither in the Y-maze experiments nor in the trap assays flies were attracted by odors of the opposite sex indicates that the chemical cues involved in *D. melanogaster* sexual behavior are not sufficient to attract flies even over the short distances tested.

## Discussion

Vinegar flies are known to exhibit social behaviors and respond to conspecifics, but most studies investigating these responses have focused on contact assays (Gil-Martí et al. 2023). The importance of contextual factors in odor-guided behavior is now increasingly recognized, and recent studies have begun to focus on olfactory responses under airflow and distance-dependent conditions (Mills et al. 2026; Stupski and Breugel 2024). When tested for long-distance effects of fly-emitted odors, experiments usually involved pure compounds instead of the natural headspace of flies. For instance, the well-characterized fly pheromone 11-cis-vaccenyl acetate (cVA) has been shown to attract both male and female flies in wind tunnel assays (Bartelt et al. 1985) and in the flywalk assay (Steck et al. 2012), where individual walking flies respond to puffed odorants in a constant airflow. Similarly, female *Drosophila melanogaster* emit Z-4-undecenal, a compound that, when tested as a pure odorant, attracts both sexes in a wind tunnel assay (Lebreton et al. 2017). In addition, it was found that food which was exposed to flies before became more attractive to conspecific flies (Cazalé-Debat et al. 2019; Wertheim et al. 2006), showing that flies can label food with compounds that attract other flies over distance. Here we ask whether flies themselves (i.e., not their olfactory remains at feeding places) attract other flies by olfactory cues. However, in this case, we did not observe such clear attraction responses when testing the headspace of living flies. Specifically, females did not show attraction to the headspace of males, despite the presence of cVA, and neither males nor females were attracted to female headspace. This suggests that fly-derived odorants that can elicit attraction as isolated compounds may not be sufficient to drive attraction to the headspace of living flies.

Our finding that females were not attracted to male headspace aligns with previous work showing a lack of attraction in the flywalk assay (Steck et al. 2012). However, our results diverge from that study’s findings regarding male responses. While Steck et al. (2012) reported significant attraction of males to both male and female headspaces, we observed no response to female headspace and even aversion to male headspace in the Y-maze. Since our carbon dioxide analysis revealed no elevated CO₂ levels in the fly headspace (Fig. 1C), it is likely that the observed aversion was due to other male-derived odors rather than CO₂. Although cVA has been described as an aggregation pheromone that governs attraction in the presence of food odors (Bartelt et al. 1985), it appears that the concentration of cVA in the headspace of living males was insufficient to induce attraction under our experimental conditions.

Olfactory cues are well known to drive courtship behavior in *D. melanogaster* (Amrein 2004; Dweck et al. 2015; Lebreton et al. 2015). Therefore, we initially expected to observe attraction of flies towards the headspaces of potential mating partners. However, most studies demonstrating the role of pheromones in courtship have used short-range assays where flies also had access to visual and auditory signals of potential mates. The absence of such multisensory cues in our setups may explain the lack of attraction. Our findings suggest that olfactory signals alone may have a relatively short functional range and that successful mate-seeking behavior likely relies on a combination of sensory inputs.

We then investigated whether social isolation could modulate flies’ behavioral responses to headspace odors. Social isolation has been shown to influence a wide range of behaviors in *Drosophila*, including learning and aggression (Vora et al. 2022). For instance, isolated males show increased aggression upon acute exposure to other males compared to socially reared males (Hoffmann 1990). At the same time, social isolation can modify the flies’ physiological and behavioral responses to fly-derived odorants (Liu et al. 2011; Sethi et al. 2019; Vora et al. 2022). Another study revealed that male flies responded differentially to cVA when they perceived this compound during their development (Tolassy et al. 2023). Given these contrasting findings, we did not have a clear expectation regarding the effect of isolation on headspace attraction. Surprisingly, even under these conditions, we observed no significant attraction or aversion to headspaces of either sex. Additionally, isolated flies exhibited delayed decision-making, and this was even more pronounced in the isolated males (Supplementary Fig. 1). This suggests that social isolation may reduce overall responsiveness to olfactory cues. Previous studies have reported increased aggression toward conspecifics in socially isolated flies (Vora et al. 2022). Such heightened aggression might predict a faster approach toward other flies. However, our results instead indicate slower decision-making, suggesting that social isolation may impair the processing or evaluation of olfactory cues rather than simply altering motivational states. In this study, social isolation was imposed only after adult eclosion, allowing us to specifically assess the contribution of adult social experience to odor-guided behavior, while not addressing potential effects arising from exposure to conspecific cues during embryonic or larval development (Cortot et al. 2022). Disentangling the contributions of early developmental exposure versus adult social experience will be an important direction for future work.

We also hypothesized that virgin flies, particularly females, might exhibit attraction to the headspace of conspecifics in the presence of food odors, given that a long-range female pheromone has been identified and shown to elicit increased responses in a food odor background (Borrero-Echeverry et al. 2022). Previous studies have reported synergistic effects between food odors and cVA, enhancing long-range attraction (Bartelt et al. 1985; Lebreton et al. 2015; Wertheim et al. 2006) and inducing mating behavior (Das et al. 2017). Despite these reports, our experiments using fly headspace in the presence of food odors did not show any significant attraction to male or female headspaces (Fig. 1F). This suggests that under the tested conditions, the presence of food odors alone was insufficient to enhance fly-derived odor attraction.

Flies seem to be able to attract other flies via labeling food with attractive substances (Cazalé-Debat et al. 2019; Wertheim et al. 2006). As the headspace of our flies did not result in similar attraction, the described labeling effect might be due to an accumulation of compounds over time that finally results in stronger attraction. An alternative explanation might be that flies actively label food, i.e. actively release fly scents that hence, in our headspace of flies that had no direct access to food, were not present.

In nature, *D. melanogaster* typically meet and mate on rotting fruit. While fly-derived odours, including pheromones, undoubtedly play a role in governing mating behavior once flies arrive on a substrate, our findings suggest that these odours may have a limited role in attracting flies on their own. It is more likely that *D. melanogaster* find each other mainly by targeting food odours, probably including those added by the flies when they label the food. Fly odours emitted directly by the flies seem to become relevant only at shorter distances, when other modalities like vision and sound also come into play. 

## Supplementary Information

Supplementary Material 1.

Supplemantary Material 2. Supplementary Figure 1. Comparison of time taken to make a choice between isolated and group-reared flies. *** indicate *p* < 0.001 in Tukey adjusted post hoc test of glm with sex x rearing condition for positive skewed data (of time).

## Data Availability

No datasets were generated or analysed during the current study.
