# Supplementary material for: Drosophila melanogaster Fly Odors Alone are not Sufficient to Attract other Flies
Source: J Chem Ecol. 2026 Aug 6;52(4):72. doi: 10.1007/s10886-026-01747-x (PMC13447572; doi:10.1007/s10886-026-01747-x)

Supplementary Figure 1. Comparison of time taken to make a choice between isolated and group-reared flies. *** indicate $p < 0.001$ in Tukey adjusted post hoc test of glm with sex x rearing condition for positive skewed data (of time).

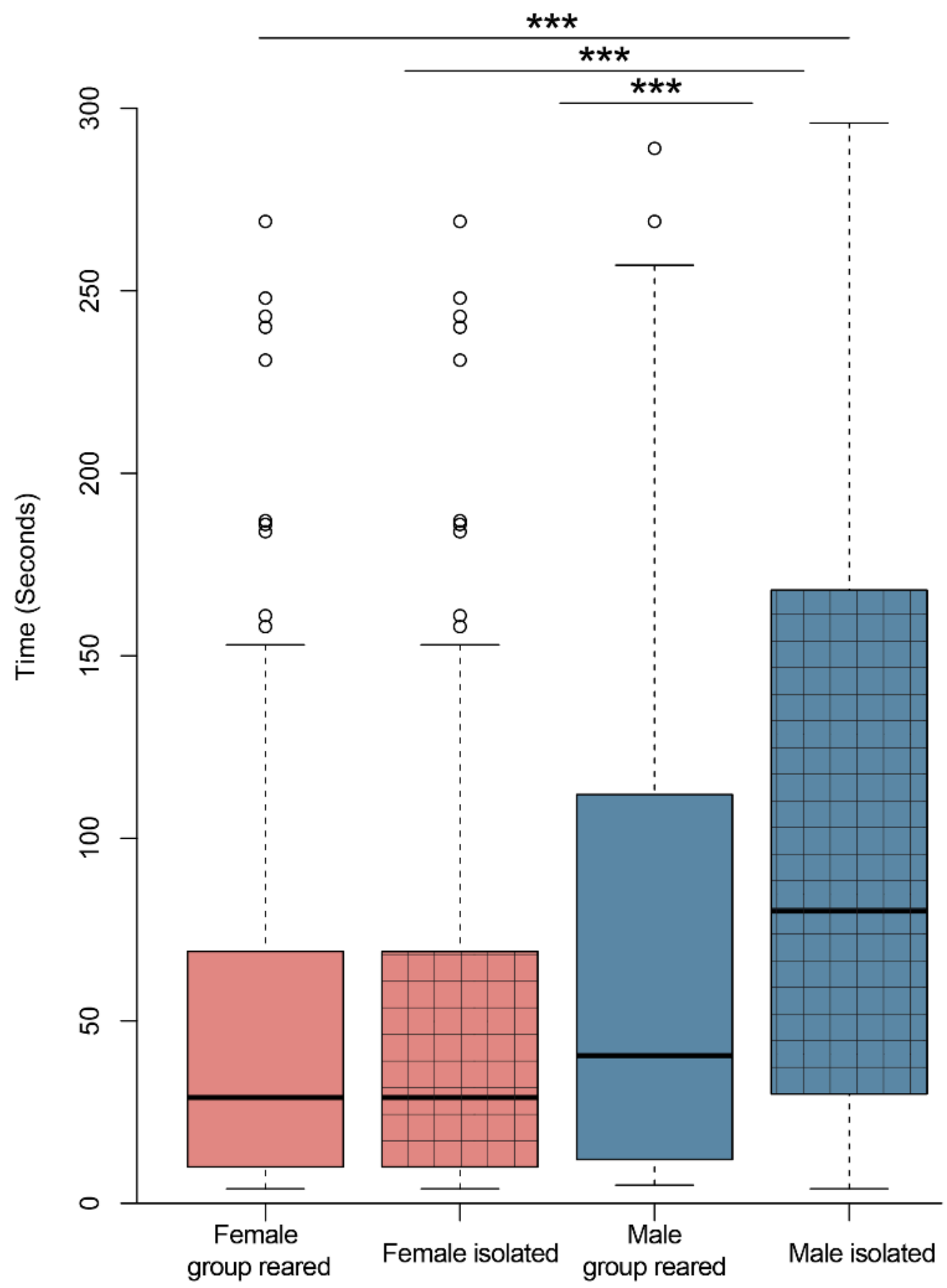

Supplement: Supplementary file 1 [file 10886_2026_1747_MOESM2_ESM.pdf]
